# Assessing virtual education on nurses’ perception and knowledge of developmental care of preterm infants: a quasi-experimental study

**DOI:** 10.1186/s12912-022-00939-6

**Published:** 2022-06-23

**Authors:** Shahla Jalali, Behnaz Bagherian, Roghayeh Mehdipour-Rabori, Mansooreh Azizzadeh forouzi, Callista Roy, Zahra Jamali, Monirsadat Nematollahi

**Affiliations:** 1 Pediatric and neonatal intensive nursing department, Razi nursing and midwifery faculty, Kerman University of medical science, Kerman, Iran; 2 Nursing research center, Kerman University of Medical Sciences, Haft Bagh Alavi highway, Kerman, Iran; 3 Mount Saint Mary’s University Los Angeles, Los Angeles, CA USA; 4 Connell School of Nursing, Boston, MA USA; 5 Department of Pediatrics, School of Medicine, Afzalipour Hospital, Kerman University of Medical Sciences, Kerman, Iran

**Keywords:** Preterm infants, Virtual education, Nurse, Neonatal intensive care unit, Developmental care

## Abstract

**Background:**

To implement developmental care accurately, neonatal intensive care unit nurses should have a proper understanding and sufficient knowledge in this field. Applying new approaches in education such as offline and online education help nurses improve their skills and knowledge. This study aimed to investigate the effect of virtual education on the perception and knowledge of neonatal developmental care in nurses working in neonatal intensive care units.

**Methods:**

This quasi-experimental study was conducted using a pretest-posttest design with two groups. The participants were 60 nurses working in neonatal intensive care units who were selected using convenience sampling (30 persons in each group). The data were collected before and 1 month after the intervention. The participants in the intervention group received developmental care training using an electronic file uploaded to Navid Learning Management System, while the members of the control group received no intervention. The instruments used to collect the data were the Demographic Information Questionnaire, the Developmental Care Knowledge Scale, and the Developmental Care Perception Scale. The collected data were analyzed using SPSS V25 software. All statistical tests were performed at the significance level of 0.05.

**Results:**

The Developmental Care perception scores before the intervention in the control and intervention groups were 83.40 ± 11.36 and 84.53 ± 9.48, respectively, showing no statistically significant difference (*P* = 0.67). Also, Developmental Care perception scores after the intervention in the control and intervention groups were 83.16 ± 13.73, and 94.70 ± 6.89, respectively, showing a statistically significant difference (*P* < 0.001). The results of paired t-test showed that the mean knowledge score in the control group before and after the intervention was not statistically significant (*P* < 0.903), while in the intervention group there was a statistically significant difference between the mean knowledge score before and after the intervention (*P* < 0.001).

The Developmental Care Knowledge scores before the intervention in the control and intervention groups were 52.66 ± 18.08 and 77.16 ± 17.20, respectively, showing a statistically significant difference (*P* = 0.001). Also, Developmental Care Knowledge scores after the intervention in the control and intervention groups were 53.66 ± 26.55and 90.33 ± 13.82, respectively, showing a statistically significant difference (*P* < 0.001). The results of paired t-test showed that the mean knowledge score in the control group before and after the intervention was not statistically significant, while in the intervention group there was a statistically significant difference between the mean knowledge score before and after the intervention.

**Conclusion:**

The results of this study showed that virtual education for the developmental care of premature infants plays an effective role in the perception and knowledge of nurses working in the neonatal intensive care unit. Therefore, the development of e-learning packages for developmental care and their availability for nurses can be a step to improve the quality of nursing care for infants admitted to the NICU.

## Introduction

Since premature infants are born before the due date, they need special medical care and support**.** Also, some of them should be hospitalized in neonatal intensive care units [1]. These infants are sensitive to new conditions after birth, and they expose to problems in adapting to extrauterine life [2]. Performing medical interventions in many situations saves the lives of premature infants, but these interventions are not without complications and may cause acute or chronic problems in premature infants [3]. Moreover, pain, stress, and separation from parents together with environmental stimuli (ventilators, monitors, incubators, telephone ringtones, and the sound of taps, shelves, and cupboard doors), and multiple care in the neonatal intensive care unit (NICU) can also negatively affect the infant’s health, leading to changes in heart rate and oxygen saturation levels, expanding fluctuations in blood pressure, increasing restlessness in the short run, and delaying the development of the central nervous system and brain in the long run [1, 4]. These conditions are very difficult for the newborn to tolerate [4]. To reduce the complications of neonatal admission to the NICU, quality care by the nurse is essential, and effective nursing practices are one of the most important steps for neonatal development and survival [5].

Developmental care refers to the care provided in the neonatal intensive care unit to reduce environmental stress and improve the infant’s adaptation to extrauterine life by the care and treatment team with the parents’ cooperation and empowerment [6]. This type of care is the implementation of supportive techniques such as reducing environmental stimuli (light, noise, etc.), flutter sucking, music and touch, kangaroo care, and infant pain control techniques [7]. Also, Developmental care aims to reduce the infant’s stress, store the infant’s energy, enhance the infant’s natural growth, and improve quality sleep conditions for the infant [8]. Pena (2016) showed that developmental care interventions include support for the infant with the infant’s reading behavior, measures to promote the infant’s physiological stabilization, and inducing the infant’s normal growth and maturity [6]. Razavi Nejad (2017) also emphasized the implementation of new care techniques, including symptom-based care and clustering care in implementing developmental care [9].

Implementing developmental care has both short-term and long-term consequences. Positive outcomes of developmental care include early breastfeeding and weight gain [10], shortening of infants’ hospital stay, increased parental satisfaction, and improving the child’s development in later life as one of its long-term consequences [11]. Applying music therapy as a part of developmental care led to better oxygenation of the infant and possibly increases brain development [4]. Also, the positive effects of a combination of kangaroo care and music interventions on the vital signs of the infants, relationship with neonatal behavioral development, weight gain, and in the neonatal intensive care unit and even after discharge were highlighted [4, 12]. In pain management, a study reported that educating nurses and physicians about non-pharmacological control of pain in infants by sucrose and swaddling was useful [7]. But Sabaghi ​​et al. (2016) found that the implementation of developmental care has no effect on the duration of hospitalization of infants under mechanical ventilation [8]. Since nursing premature infants admitted to the neonatal intensive care unit requires sensitivity, accuracy, skill, and experience, and given that nurses are the primary caregivers of hospitalized infants, their slightest malpractice can cause irreparable harm to the infant. Therefore, standard care training can improve the quality of nursing services and minimize infants’ physical, psychological, and developmental complications.

Currently, it is possible to use virtual education techniques to increase learners’ knowledge and skills by using appropriate technology and facilitating the learning process at any time and place [13, 14]. To make nurses familiar with the latest developments in providing care to patients, the use of technology in education is necessary [15, 16]. The advantages of e-learning include lower cost, more flexibility, ease of access, inclusiveness, and the ability to self-control in learning and adapting to individual learning goals [11]. Other advantages of e-learning are paying attention to learners’ needs, ease of access to various resources, continuous monitoring of academic achievement, and presentation and preparation of various educational models [16]. Increasing nursing knowledge and care skills improve nurses’ job satisfaction, abilities in time management [17], and the quality of services provided by them [14]. Also, Kadivar et al. (2017) stated that e-learning programs are effective in increasing the motivation of health care staff in providing neonatal care [14].

Issues such as understanding and awareness of the type of care are likely to affect its implementation. Education is one of the ways that can affect nurses’ awareness [18]. Since very limited studies have addressed virtual education for developmental care in Iran, this study aims to examine the effect of a developmental-care-based virtual education program on the perception and knowledge of nurses working in the neonatal intensive care units of Afzalipour Hospital in Kerman in 2020.

## Methods

### Research design and setting

This interventional (quasi-experimental) study was performed with a pretest-posttest design. Sixty nurses working in the neonatal intensive care units of Afzalipour Hospital in Kerman participated from January to May 2020.

Afzalipour Hospital in Kerman has two neonatal intensive care units with more than 100 beds (each ward contains 50beds) that provide services to infants at level 3A. This hospital is the first and largest educational and medical center in southeast Iran and one of the advanced centers for providing medical services and cares to infants in the south and east of Iran.

#### Sampling method

Sampling was done by the census method. First, all nurses working in the neonatal intensive care unit, who was 70, were registered. Ten nurses who did not meet the inclusion criteria were excluded from the study and thus the final sample included 60 nurses who participated in all stages of the study. Then, they were divided into control and intervention groups by simple random sampling using a random number table.

The inclusion criteria were having at least a degree in nursing and working at least 1 month in the neonatal intensive care unit. The exclusion criteria were Failure to complete at least 10% of the questionnaire and not cooperating in seeing the uploaded electronic content in Navid system within the specified time (2 weeks). In the current study, all of the participants completed the intervention, and no participants were excluded from the analysis.

#### Intervention group procedure

After obtaining the necessary permits and providing some information about the objectives of the study, written consent was received from the nurses. Then, the nurses who met the inclusion criteria were enrolled in the study using the census method. Before conducting the intervention, a meeting was held for the participants in the two groups to make them familiar with the Navid system. After making arrangements with the system administrator, each nurse was given a username and password. Then, the nurses in the two groups completed the three questionnaires including Demographic Information Questionnaire, the Developmental Care Knowledge Scale, and the Developmental Care Perception Scale.

The nurses in the intervention group received e-learning content containing instructions on neonatal developmental care, which was uploaded to the Navid System (special software for university education). The training package covered all topics on neonatal developmental care, which were extracted from the latest neonatal intensive care nursing textbooks (Table 1). To enhance the efficacy of the learners’ learning, electronic content was supplemented with instructional videos, slides, photos, and short passages with audio instructions. The training content was confirmed by two professors at the Department of Pediatrics of Kerman University of Medical Sciences (one of them was a neonatologist and another one was a ph. D in nursing). Navid System is the software system of Iran’s Ministry of Health and Medical Education and provides e-learning facilities including all services related to education, evaluation, interaction, planning, and educational management.

**Table 1:** The contents of the training program

Session	Description
1	Defining developmental care and its importance Improving the physical space in the developmental care program including facilities, access routes, corridors, and entrances of the neonatal intensive care unit, specialized hospitalization, and neonatal care space, essential support spaces in the neonatal intensive care unit The effect of excessive sensory stimulation in infants: The importance of adjusting the lighting in the NICU ‌to protect the premature infants’ vision, considerations related to the care of the preterm infant hearing, controlling the noise, the importance of developing the baby’s olfactory and taste senses, the importance of infant tactile sensation, and tips about infant massaging
2	Physical-behavioral reactions of premature infants, including behavioral evaluation and control, completing the objective observation sheet, forms for observing and recording infant behavior, identifying infant behaviors, autonomic nervous system behaviors, skin color, movements due to immaturity of the autonomic nervous system, autonomic and visceral systems, neonatal movements, specific movements of limbs, behaviors related to attention and interaction, behaviors showing self-regulatory balance, behaviors indicating neonatal stress
3	Characteristics of premature infant sleep-wake-up patterns, including the importance of sleep-wake in preterm infants, diagnosis of premature infant sleep-wake states, types of premature infant sleep-wake states, benefits of sleep in infants, complications of sleep deprivation in infants, factors disrupting premature infant sleep-state, the infant sleep space in NICU, developmental care to support the sleep of premature infants
4	Assessing the status of the infant including the importance of supporting the infant’s body and positioning the premature infant, developmental complications due to lack of support and positioning of the premature infant in the NICU, developmental benefits of positioning the preterm infant admitted to the NICU, general principles of premature neonatal positioning in the NICU, the infant’s position during kangaroo care, the principles of lifting and moving the infant NIDCAP program: clustering and symptom-based care Management and control of pain in premature infants: infant response to pain, non-pharmacological care, prescribing medication for pain control, infant pain management protocol (care before the onset of painful procedures, care during painful procedures, care about painful procedures)
5	Premature infant nutrition: oral feeding with developmental care approach, developmental care in infant feeding with a gastric tube, infant oral feeding, successful oral sucking, development of swallowing process, significant cases for diagnosing the time of oral feeding, environmental factors affecting infant oral feeding and its continuity, oral feeding patterns based on infant demand, the importance of infant hospitalization space in supporting breastfeeding, non-oral sucking
6	Family-centered care and its effects on premature infants: significant ethical issues regarding the infant and parents, success in family-centered care, family-centered care in lien with the mission of the neonatal intensive care unit, guide to social interactions with the family care group, parental education, parental support resources, mother’s residence, and infant visit rules, facilities needed for mothers’ residence

The e-learning package used in this study contained 6 separate sections. The participants had to review all content in each section so that they could go to the next step. Each participant could watch the instructional materials as many times as needed. The participants should study the materials for 2 weeks. The intervention was completed in 6 days. During the study, the researcher could have full control over the number of times the training file was viewed by the participants. Moreover, all the participants’ questions were recorded by them in the system and answered by the researcher. The system provided the possibility of two-way interaction via texting and audio and video calls between the researcher and the participants.

training files were available 24 hours a day for the participants. Given the nurses’ multiple and busy working shifts, they could view the training files at any time. Since the nurses could forget to watch the uploaded files, a reminder text message was sent to them every day. Moreover, the researcher could view the learners’ profiles in the Navid system, and the researcher could monitor the time and number of times each file was viewed by the nurses.

One month after the completion of the intervention, the participants in the intervention group again completed the questionnaires in the Navid system. To prevent the exchange of information between the members of the intervention and control groups, the participants in the intervention group were asked not to share the information with the participants in the control group until the end of the study.

#### Control group procedure

Nurses in the control group did not receive any organized training in developmental care. After making arrangements with the system administrator, each nurse of the control group was given a username and password. They completed the questionnaires before and 1 month after the intervention in the Navid system. The researcher (MSc student in neonatal intensive nursing) sent a reminder text message to them for complete questionnaires in Navid system. Upon the completion of the study, the content of the e-learning program was provided to nurses in the control group.

#### Instruments

The data in this study were collected using three instruments the Demographic Information Questionnaire, the Developmental Care Knowledge Scale, and the Developmental Care Perception Scale.

##### The demographic information questionnaire

This questionnaire assessed the participants’ gender, age, marital status, education, work experience in the neonatal intensive care unit, work shifts, position, and type of employment [19].

##### The developmental care perception scale

This tool was developed by Baghlani et al. (2019). It contains 20 items designed on a 5-point Likert scale ranging from 1 to 5. Perception statements were scored as “I completely agree”, “I agree”, “No idea”,” I disagree “, and” I completely disagree”. The total score on the scale ranges from 20 to 100 and each item is scored separately. The content validity of the scale was also assessed were assessed concerning the opinions of 10 nursing experts and neonatologists and its reliability was reported at 0.92 using Cronbach’s alpha coefficient [19].

##### The developmental care knowledge scale

The scale was developed by Baghlani et al. (2019). The questionnaire consisted of 20 four-option questions, with each question having one correct answer receiving one score, whereas the other options were considered incorrect answers receiving a zero score. To make a better comparison, the range of scores was reported from 0 to 100. The validity of the instrument was assessed concerning the opinions of 10 nursing experts and neonatologists. Besides, its reliability was estimated using Cronbach’s alpha reported 0.70 which indicated the acceptable reliability of the scale [19].

#### Data analysis

To analyze the data, SPSS 25 (SPSS Inc., Chicago, Ill., USA) was used. Descriptive statistics (percentage, frequency, mean, and standard deviation) were applied to describe the demographic characteristics of the participants’ study. Chi-square and Fisher’s exact tests were also used to compare the similarity of the intervention and control groups in terms of demographic variables. Based on the Kolmogorov-Smirnov, independent samples t-test was used to compare the scores of Developmental Care knowledge and perception between the two groups at the pre-and post-intervention stages. Paired t-test was also administered to compare Developmental Care knowledge and perception scores in each group at pre-and post-intervention stages. Analysis of covariance was applied to control the impact of the pretest on the scores of neonatal Developmental Care knowledge and perception. The significance level was set at *P* < 0.05.

## Results

The participants in this study were two groups of female nurses (*n* = 60) working in neonatal intensive care units. Most of the nurses in the intervention (56.7%) and control group (73.3%) were 20–30 years old. Moreover, there were no statistically significant differences between the two groups in terms of demographic characteristics such as age, marital status, having a premature infant, education, having a nursing degree, employment, working shift, attendance in training courses, and work experience. In other words, the two groups were homogenous in terms of demographic variables (*p* < 0.05) (Table 2).

**Table 2:** The participants’ demographic characteristics of nurses in the intervention and control groups

Variable	Categories	Intervention (*n* = 30)		Control (*n* = 30)		Results
		Number	%	Number	%	
Age (years)^a^	20–30	17	56.7	22	73.3	**χ**^**2**^**(2) = 5.496** ***P*** **= 0.064**
	31–40	4	13.3	6	20	
	41–50	9	30	2	6.7	
Marital status^a^	Single	12	40	11	36.7	**χ**^**2**^**(1) = 0.071** ***P*** **= 0.791**
	Married	18	60	19	63.3	
Premature child^b^	Yes	1	3.3	0	0	1P =
	No	29	96.7	30	100	
Education^a^	Bachelor’s degree	25	83.3	24	80	**χ**^**2**^**(1) = 0.111** ***P*** **= 0.739**
	Master’s degree	5	16.7	6	20	
Field of study^a^	Pediatrics	5	16.7	7	23.3	**χ**^**2**^**(1) = 0.417** ***P*** **= 0.519**
	Nursing	25	83.3	23	76.8	
Employment^a^	Official	11	36.7	10	33.3	**χ**^**2**^**(2) = 0.698** ***P*** **= 0.705**
	Contractual	9	30	12	40	
	Plan-based	10	33.3	8	26.7	
Work shift^b^	Fixed	1	3.3	2	6.7	1P =
	Rotating	29	96.7	28	93.3	
Participation in training courses	Yes	5	16.7	10	33.3	**χ**^**2**^**(1) = 2.222** ***P*** **= 0.136**
	No	25	83.3	20	66.7	
Service records in NICU (year)^a^	1>	10	33.3	5	16.7	**χ**^**2**^**(2) = 4.602** ***P*** **= 0.100**
	1–5	9	30	17	56.7	
	< 5	11	36.7	8	26.7	

^a^Chi-square test and

^b^Fisher’s exact test

Before the intervention, there were no significant differences in the mean scores of neonatal developmental care perception between the intervention group (84.53 ± 9.48) and the control group (83.40 ± 11.36) as indicated by the independent samples t-test (*P* = 0.67).

However, the results of the independent t-test showed significant differences in the mean scores of neonatal developmental care perception between the intervention group (94.70 ± 6.89) and the control group (83.16 ± 13.73) after the intervention (*P* < 0.001), indicating a significant increase in the mean score of neonatal developmental care perception in the intervention group compared to the control group (Table 3).

**Table 3:** comparing the mean scores for participants’ perception of neonatal developmental care inter and between two groups

Developmental care perception	group	Pre-intervention^b^	Post-intervention^a^	Mean difference	Intragroup comparison^c^
Neonatal	Intervention	51.73 ± 5.87	56.80 ± 4.14	−5.066	t (29) = −4.630 *P* < 0.001
	Control	50.43 ± 7.22	50.40 ± 8.52	0.033	t (29) = 0.028 *P* = 0.977
	Mean difference	1.30	6.40	–	–
	Intergroup statistics	t(58) = 0.765 *P* = 0.447	F(1,57) = 15.233 *P* < 0.001 Ƞ^2^ = 0.211	**–**	**–**
Nursing	Intervention	32.80 ± 5.01	37.90 ± 3.33	−5.100	t (29) = − 4.833 *P* < 0.001
	Control	32.96 ± 5.54	32.76 ± 5.55	0.200	t (29) = 0.262 *P* < 0.796
	Mean difference	−1.166	5.14	–	
	Intergroup statistics	t(58) = −0.135 *P* = 0.893	F(1,57) = 23.054 *P* < 0.001 Ƞ^2^ = 0.288		
Developmental care perception	Intervention	84.53 ± 9.48	37.90 ± 3.33	−10.66	t (29) = − 5.381 *P* < 0.001
	Control	83.40 ± 11.36	32.76 ± 5.55	0.233	t (29) = 0.123 *P* < 0.903
	Mean difference	1.133	5.14	–	
	Intergroup comparision	t(58) = 0.419 *P* = 0.677	F(1,57) = 23.054 *P* < 0.001 Ƞ^2^ = 0.288		

^a^Univariate analysis of covariance

^b^Independent samples t

^c^Paired samples t-test, Ƞ^2^ effect size

The covariance analysis test was run to control and investigate the effects of the pretest on nurses’ scores of neonatal Developmental Care perception. The results showed a statistically significant difference between the control and intervention groups in the total post-test scores of neonatal Developmental Care perception. The increase in the mean score of neonatal developmental care perception in the intervention group is much greater than in the control group after the intervention. Cohen’s d showed an effect size of 2.88. Thus this difference is relatively high in the community.

The results also suggested that there were significant differences in the mean scores of neonatal developmental care knowledge between the intervention group (77.16 ± 17.20) and the control group (52.66 ± 18.08) before the intervention as indicated by the independent samples t-test (*P* < 0.001). Furthermore, the results of the independent t-test showed significant differences in the mean scores of neonatal developmental care knowledge between the intervention group (90.33 ± 13.82) and the control group (53.66 ± 26.55) after the intervention (*P* < 0.001), indicating a significant increase in the mean score of neonatal developmental care knowledge in the intervention group compared to the control group (Table 4). Based on the results of paired t-test, the scores of neonatal developmental care knowledge increased by 1 and 13.16 points in the control and intervention groups 1 month after the intervention, respectively. The increase in the mean score of neonatal developmental care knowledge in the intervention group is much greater than in the control group after the intervention. Cohen’s d showed the effect size 0.168. Thus this difference is relatively high in the community.

**Table 4:** Comparing the mean scores for participants’ knowledge of neonatal developmental care inter and between two groups

Group	Pre-intervention^b^	Post-intervention^a^	Mean difference	Intragroup comparison^c^
Intervention	77.16 ± 17.20	90.33 ± 13.82	−13.16	t (29) = − 4.111 *P* < 0.001
Control	52.66 ± 18.08	53.66 ± 26.55	−1	t (29) = − 0.379 *P* = 0.702
Mean difference	24.50	36.67		
Intergroup comparison	t(58) = 5.376 *P* = 0.001	F(1,57) = 11.478 *P* < 0.001 Ƞ^2^ = 0.168		

^a^Univariate analysis of covariance

^b^Independent samples t

^c^Paired samples t-test, Ƞ^2^ effect size

## Discussion

The present study investigated the effect of virtual education on the perception and knowledge of neonatal developmental care in nurses working in neonatal intensive care units. The results indicated that the training program increased nurses’ perception and knowledge about neonatal developmental care significantly and with a very large effect size. A review of the literature showed that there was no study addressing the effect of e-learning for the developmental care of premature infants on the perception of nurses working in neonatal intensive care units. Thus, this section reviews studies that had a greater similarity with the present study. Similar to the study, Khatiban et al. (2014) reported that education with conventional approaches had a positive effect on the perception of intensive care nurses about the barriers to compliance with general infection control standards [20]. whereas in the present study, the nurses received offline training with an electronic package uploaded in Navid system. In applying e-learning, in addition to educational content, the proper quality of software used is very important and Navid software was one of the highest quality university education software in Iran. Also, It provided e-learning facilities including all services related to education, evaluation, interaction, planning, and educational management.

In another study, Solhaug (2010) measured the effect of the use of developmental care on nurses’ perceptions in an intensive care unit and concluded that nurses’ perceptions increased after the implementation of the developmental care program [11]. This study measured the impact of developmental care on nurses’ understanding but it differed from the current study in that the intervention was not conducted in the form of a training program.

The researchers of the current study prepared the content of e-learning in the form of videos, photos, and slides with lectures, and all the above items were prepared from the real environment of the neonatal intensive care unit, it was able to have a positive effect on the nurses’ knowledge and perception.

A descriptive study by Mosqueda-Pena (2016) showed the positive effect of developmental care training on the knowledge and satisfaction of 566 health care professionals in neonatal intensive care units [6]. This study was similar to the present study in terms of developmental care training but differed from the present study in terms of the type of training course implemented. The authors used traditional training methods and workshops to provide training to the participants. In the current study, the electronic content was available to nurses for 2 weeks and they could watch it at any time of the day or night. There was also no limit to the number of views of the educational content. Perhaps one of the reasons for the impact of offline training in the current study is the flexible nature of the intervention time.

Zamani et al. (2019) examined the effect of web-based education on NICU nurses’ knowledge, attitude, and care performance and showed that the average knowledge score in the web-based education group increased significantly compared to the control group. However, there was no significant difference between the two groups in terms of mean scores of attitude and care performance [21]. In this study, the educational content was instructed in the form of general care in the neonatal intensive care unit. Whereas, the training content in the present study focused on developmental care for premature infants.

Presenting content in a specialized and limited way to a specific care topic may be one of the strengths of the current study.

Nevertheless, both studies used e-learning techniques. Web-based education seems to not only update nurses’ care information but also teach them how to use new technologies. Since it was assumed that not all nurses are at the same level to work with the software, in the current study, to familiarize nurses with the software, a training session was scheduled for both groups at the beginning of the study.

Furthermore, e-learning is very suitable for people who have developed self-discipline, and nurses can participate in such training courses by managing their time based on their working shifts in the hospital. During the current study, none of the participants were excluded due to missing the training course.

Deloian (2015) showed that web-based training significantly increased NICU nurses’ knowledge of breastfeeding after the intervention [22]. This finding was similar to the present study in terms of the effectiveness of e-learning courses. it seems that the preparation of educational content according to the needs of individuals should also be considered. The studied nurses should have the knowledge and skills of developmental care to be able to implement it in NICU.

Agrawal et al. (2016) examined the effectiveness of virtual classroom training in improving the knowledge and key maternal neonatal health skills of general midwifery students and found that virtual classroom training was effective in improving students’ knowledge and key skills [23]. In the present study, nurses ‘knowledge and perception of neonatal developmental care were assessed, but unlike the above study, measuring nurses’ performance was not the aim of the study.

Hasanpour et al. (2017) studied the effect of a neonatal sleep care training program on nurses’ knowledge and practice in neonatal intensive care units and showed that training increased nurses’ knowledge, but there was no significant change in their performance [24].

Although the performance of nurses was not evaluated in the current study, to evaluate the effect of performance, it was necessary to follow the study for a longer period. Follow-up studies should be done in 3 and 6-month intervals. Therefore, it is essential to organize and implement long-term training programs.

In addition, the training content in Hasanpour et al.’s (2017) study focused on infants’ sleep and wakefulness, which is one of the goals of developmental care. Similarly, the present study addressed infants’ sleep and wakefulness as part of the educational content of developmental care.

Given the importance of developmental care in limiting complications in premature infants, the implementation of such training is also useful to improve nurses’ care skills since raising nurses’ awareness and knowledge also leads to increasing the quality of care performance. In their review study, Als et al. (2011) highlighted the importance of educating nurses in the newborn individualized developmental care and assessment program (NIDCAP) with kangaroo mother care (KMC) because the implementation of these training programs not only increases nurses’ knowledge in a given field but also improves their awareness of the consequences of their implementation, e.g. their effect on the development of infants’ brain function and central nervous system [25]. Offering developmental care by nurses in the neonatal intensive care unit leads to quality care, and infants are discharged with minimal complications. Thus, nurses must have sufficient knowledge and understanding of the type of care to be able to demonstrate proper care performance. On the other hand, the use of virtual education and e-learning techniques according to nurses’ working conditions is one of the most effective strategies for empowering nurses.

### Limitations

This study had some limitations that need to be observed. A self-report questionnaire was administered to measure the effectiveness of an educational program in improving the nurses’ knowledge and perception in neonatal developmental care. Assessment of perception and knowledge of neonatal developmental care might have been affected by the participants’ bias inherent in the self-report questionnaire. In other words, since nurses may have tended to overrate their levels of knowledge and perception, the data might not reflect the actual level of nurses’ knowledge and perception. Future studies can use objective rather than self-reported means of evaluating knowledge and perception and blended methods of competency evaluation to determine the actual level of knowledge and perception among nurses in neonatal developmental care. Finally, data collection was conducted 1 month after the intervention. Future longer follow-ups (3–6) are recommended to have more accurate results, determine the long-term impact of training, and assess the effect of educational courses on nurses.

Another limitation of this study was that the participants were selected from a single medical center that has been affected by generalization of data.

## Conclusion

The present study showed that virtual education on how to implement developmental care for premature infants has an effective role in improving the understanding and knowledge of nurses working in neonatal intensive care units. Since nurses play a key role in providing care services, it is essential to pay attention to promoting their knowledge and awareness. Virtual education is recommended as a flexible, accessible, and low-cost method that allows access to multimedia and engaging educational content anywhere and anytime to train nurses in neonatal intensive care units and other wards. Thus, officials and managers of hospitals and medical centers are recommended to make necessary planning for the implementation of virtual education for nurses working in neonatal intensive care units.

In this regard, nursing professors are recommended to conduct systematic developmental care training using new educational approaches, such as virtual (online and offline) and face-to-face teaching to compare their outcomes in clinical practice. Also, they can benefit from our experiences in administration of the online or offline training programs for nurses.

## Data Availability

The datasets used and/or analyzed during the current study are available from the corresponding author on reasonable request.
